# Prenatal and childhood chlordecone exposure, cognitive abilities and problem behaviors in 7-year-old children: the TIMOUN mother–child cohort in Guadeloupe

**DOI:** 10.1186/s12940-023-00970-3

**Published:** 2023-02-27

**Authors:** Youssef Oulhote, Florence Rouget, Léah Michineau, Christine Monfort, Mireille Desrochers-Couture, Jean-Pierre Thomé, Philippe Kadhel, Luc Multigner, Sylvaine Cordier, Gina Muckle

**Affiliations:** 1 Department of Biostatistics and Epidemiology, School of Public Health and Health Sciences, University of Massachusetts, Amherst, MA 01003 USA; 2 CHU De Rennes, Univ Rennes, Inserm, EHESP, Irset (Institut De Recherche En Santé, Environnement Et Travail) - UMR_S 1085, Rennes, France; 3 Univ Rennes, Inserm, EHESP, Irset (Institut De Recherche En Santé, Environnement Et Travail) - UMR_S 1085, Rennes, France; 4 Population Health and Optimal Health Practices Research Unit, CHU De Québec Research Centre; École de Psychologie, Université Laval, Pavillon Félix-Antoine-Savard, 2325 Rue Des Bibliothèques, Québec City, Québec G1V 0A6 Canada; 5 LEAE-CART (Laboratoire d’Ecologie Animale Et d’Ecotoxicologie-Centre De Recherche Analytique Et Technologique), Université De Liège, B-4000 Liège, Belgium; 6 CHU De de La Guadeloupe, Univ Antilles, Univ Rennes, Inserm, EHESP, Irset (Institut De Recherche En Santé, Environnement Et Travail) - UMR_S 1085, Pointe-À-Pitre, France

**Keywords:** Chlordecone, Organochlorine pesticides, Cognitive abilities, Behavior, Childhood, Guadeloupe

## Abstract

**Background:**

Chlordecone is a highly persistent organochlorine insecticide that was intensively used in banana fields in the French West Indies, resulting in a widespread contamination. Neurotoxicity of acute exposures in adults is well recognized, and empirical data suggests that prenatal exposure affects visual and fine motor developments during infancy and childhood, with greater susceptibility in boys.

**Objective:**

To assess the associations between pre- and postnatal exposures to chlordecone and cognitive and behavioral functions in school-aged children from Guadeloupe.

**Methods:**

We examined 576 children from the TIMOUN mother–child cohort in Guadeloupe at 7 years of age. Concentrations of chlordecone and other environmental contaminants were measured in cord- and children’s blood at age 7 years. Cognitive abilities of children were assessed with the Wechsler Intelligence Scale for Children-IV (WISC-IV), and externalizing and internalizing problem behaviors documented with the Strengths and Difficulties Questionnaire (SDQ) completed by the child’s mother. We estimated covariate-adjusted associations between cord- and 7-years chlordecone concentrations and child outcomes using structural equations modeling, and tested effect modification by sex.

**Results:**

Geometric means of blood chlordecone concentrations were 0.13 µg/L in cord blood and 0.06 µg/L in children’s blood at age 7 years. A twofold increase in cord blood concentrations was associated with 0.05 standard deviation (SD) (95% Confidence Interval [CI]: 0.0, 0.10) higher internalizing problem scores, whereas 7-years chlordecone concentrations were associated with lower Full-Scale IQ scores (FSIQ) and greater externalized behavioral problem scores. A twofold increase in 7-year chlordecone concentrations was associated with a decrease of 0.67 point (95% CI: -1.13, -0.22) on FSIQ and an increase of 0.04 SD (95% CI: 0.0, 0.07) on externalizing problems. These associations with Cognitive abilities were driven by decreases in perceptive reasoning, working memory and verbal comprehension. Associations between 7-year exposure and perceptive reasoning, working memory, and the FSIQ were stronger in boys, whereas cord blood and child blood associations with internalizing problems were stronger in girls.

**Conclusions:**

These results suggests that cognitive abilities and externalizing behavior problems at school age are impaired by childhood, but not in utero, exposure to chlordecone, and that prenatal exposure is related to greater internalizing behavioral problems.

**Supplementary Information:**

The online version contains supplementary material available at 10.1186/s12940-023-00970-3.

## Background

Chlordecone (Kepone ™) is an organochlorine insecticide principally used for control of the banana root borer in Central and South America and the Caribbean, including Puerto Rico. This chemical was initially manufactured in the U.S. from 1951 to until 1976 when production and use were banned. Nonetheless, chlordecone under the brand name of Curlone ™ has been intensively used from 1976 to 1993 in banana fields in the French West Indies (FWI). This pesticide undergoes no significant biotic or abiotic degradation in the environment [2]. It has been estimated that the duration of chlordecone pollution of soil in FWI will last for decades to centuries [6]. Although chlordecone has not been used since 1993, it persists in the soil of current and former banana fields where it has been spread. Chlordecone in soil is slowly drained by rainfall towards superficial water, ground water, and marine coastal waters and contaminates the terrestrial and aquatic ecosystems, including crops, livestock, and fishing products [25]. Therefore, exposure to environmental levels to chlordecone have been documented in the FWI populations, including pregnant women, through consumption of contaminated foodstuffs [15, 24].

Chlordecone is a recognized reproductive and developmental toxicant, neurotoxic and carcinogenic in rodents [2]. Studies in humans with high occupational exposure to chlordecone demonstrate toxic effects on the nervous system, liver, and reproductive system. Chlordecone is an endocrine-disrupting chemical (EDC) with well recognized estrogenic properties both in vitro and in vivo [16, 23]. Consequently, it can perturb hormonal systems, and interfere with normal development during sensitive periods from conception to childhood. From our prospective Timoun mother–child cohort in Guadeloupe, we previously reported several pregnancy outcomes related to maternal exposure, including hypotensive effects [34], decreased length of gestation, and increased risk of preterm birth [20]. Prenatal exposure (cord-blood) to chlordecone was associated with decreased weight of newborns from mothers with excessive weight gain prenatally [18], but no risk of overall malformations in newborns were reported [31]. In infants from this cohort at 3-months of age, prenatal exposure to chlordecone was associated with increased thyroid stimulating hormone (TSH) levels in boys only [7]. When aged 7-months old, infants exhibited poorer visual recognition memory or novelty preference in relation with pre- and postnatal chlordecone exposures. Prenatal exposure to chlordecone was also related to slower visual processing speed [9]. We also reported associations between prenatal exposure and poor fine motor development at 7-months [9] and at 18-months of age, but in boys only [5].

Our group recently published the first results from the follow-up of the Timoun study at age 7 years, showing that in utero exposure and during childhood impairs visual contrast sensitivity in boys [33], whereas no associations were found with sex-typed toy preference playing time [8]. In this study, we investigated whether prenatal and childhood exposures to chlordecone are associated with cognitive abilities and behavioral problems in school aged children at 7 years.

## Methods

### Study population

The TIMOUN birth cohort included 1068 pregnant women during their second- or third-trimester prenatal visit recruited at public hospitals and antenatal care dispensary between 2004 and 2007 in Guadeloupe archipelago (FWI). Eligible participants resided in Guadeloupe for more than 3 years. Around 7% refused to participate mainly because of refusal of the spouse, not wishing to participate in the follow-up, and not wishing to provide biological samples [20]. Mothers answered a standardized questionnaire during a face-to-face interview at enrollment. This questionnaire included sociodemographic characteristics, occupational, medical, and obstetrical information. Gestational age (in weeks of amenorrhea) was estimated by obstetricians in charge of the follow up of the pregnancy. At delivery, dietary habits and alcohol consumption during pregnancy and newborn’s health data (including birth weight) were collected and a cord blood sample was obtained to document prenatal exposure to chlordecone and other environmental contaminants. At 7 years-old, the whole cohort of liveborn singleton children (*N* = 1033) were invited to participate in a clinical examination, 444 families could not be contacted, refused to participate, or were excluded for other reasons (Supplementary material; Figure S1). At 7-years, 576 of the participating children underwent a neuropsychological evaluation. A maternal interview provided information concerning current health and past medical history, lifestyle, duration of breastfeeding, child behavior and other characteristics. Children’s blood samples were also obtained at age 7. The study was approved by the relevant ethical committee for studies involving human subjects (Comité de Protection des Personnes Sud-Ouest et Outremer III; n° 2011-AOOSSI–40). Each parent provided written informed consent.

### Assessment of cognitive function

We assessed children’s cognitive abilities with the French validated version for France of the Wechsler Intelligence Scale for Children, fourth edition (WISC-IV) [39]. The eight core subtests required to compute the Full-Scale IQ score (FSIQ) were performed. The administration of these core subtests provides scaled scores standardized for age, which are combined to obtain four composite scores in domains of verbal comprehension (Similarities and Vocabulary), processing speed (Coding and Symbols), working memory (Letter-Number Sequencing and Digit Span), and perceptive reasoning (Block Design and Matrix Reasoning). The sum of the 8 scaled scores provides the FSIQ score, a measure of global intellectual functioning, which was our primary outcome of cognitive abilities.

### Behavior assessment

Child behavior problems were documented with the French version of the Strengths and Difficulties Questionnaire (SDQ), a 25-items screening questionnaire completed by the child’s parent. Items are representing attributes, some positives and other negatives, scored on a 3-point Likert scale: 0 (“not true”), 1 (“somewhat true”), and 2 (“certainly true”), documenting emotional symptoms, conduct problems, hyperactivity/inattention, peer relationship problems and prosocial behavior. There are strong theoretical and empirical supports for classifying behavior problems according internalizing and externalizing-types of problems. The SDQ’s emotional symptoms (5 items) and peer relationship subscales (5 items) are usually combined into an internalizing problems subscale, as are the conduct (5 items) and hyperactivity/inattention (5 items) subscales into an externalizing problems subscale [13], and higher scores indicate higher difficulties. The SDQ is adapted for many different cultures and languages and has demonstrated excellent psychometric properties (http://www.sdqinfo.com/).

### Biomarkers of exposure to chlordecone and other contaminants

Blood samples from the umbilical cord and the child at the 7-year visit were collected in EDTA tubes to document respectively prenatal and childhood exposure to chlordecone and other environmental contaminants. Plasma samples were stored at -30 °C in Polypropylene Nunc® tubes following centrifugation. Chlordecone, polychlorinated biphenyl congener 153 (PCB-153), dichlorodiphenyl dichloroethene (DDE) and lipids were measured in plasma. Total mercury (Hg), lead (Pb), and cadmium (Cd) were quantified in whole blood using inductively coupled plasma mass spectrometry. Determination of chlordecone and PCB-153 concentrations were done by the Center for Analytical and Research Technology at Liege University (Belgium). Contaminant concentrations were analyzed by high-resolution gas chromatography (Thermo Quest Trace 2000, Milan, Italy) equipped with a Ni63 electron capture detection. Preparation of samples and quantification method were previously described [24]. The limit of detection (LOD) was 0.06 μg/L for chlordecone, PCB-153, and DDE in cord blood, and 0.02 μg/L for child chlordecone, PCB-153, and DDE. Total cholesterol and triglyceride in plasma were determined by standard enzymatic procedures (DiaSys Diagnostoc Systems GmbH,Holzheim, Germany) and total lipid concentrations were calculated as described by Bernert et al. [4]. Blood Hg, Pb, and Cd concentrations were measured by inductively coupled plasma mass spectrometry (ICP-MS) at the laboratory of the Centre de toxicologie du Québec. The LOD for Hg, Pb and Cd were 0.4 μg/L, 2 μg/L and 0.1 μg/L respectively, and each run of samples included a standard. The linear calibration curve (5 to 120 pg/μl) was established with a certified chlordecone solution (Riedel-de Haën, Seelze, Germany) and a good correlation (r > 0.99) was achieved. A procedural blank, consisting of 2 ml human serum (Cambrex Bio Science, Walkersville, MD), was run with each series of 10 samples, to control the clean-up procedure. Quality control (QC) was performed by regular analyses of procedural blanks, by the injection of standard and n-hexane blanks. The QC was human serum enriched with defined concentrations of chlordecone. The chlordecone concentration in each sample and in the QC was corrected for initial sample weight, and the percentage recovery of the surrogate PCB 112. Recovery rates were always between 70 and 130% [24].

### Covariates and potential confounders

We collected socio-demographic and lifestyle factors and medical history at enrollment, after delivery, and at subsequent follow-ups via administered questionnaires. We used directed acyclic graphs (Supplemental Material; Figure S2) to identify a set of covariates to include in the models from the following potential covariates: child’s age at testing (in months), sex (male; female), birth weight (grams), maternal age at pregnancy, parity (nulliparous; primipara and multiparous), duration of breastfeeding (no breastfeeding; ≤ 6 months; 7–18 months; ≥ 18 months), maternal marital status (married or in a couple; single; living with own family), socio-economic status (SES) indicators during pregnancy based on maternal education (none or some elementary school; some high school; high school diploma; college/university studies) and monthly household income (≤ 800 Euros; 800 – 2300 Euros; > 2300 Euros), maternal nonverbal cognitive abilities (Raven score) assessed with the Raven’s Progressive Matrices [29], maternal alcohol consumption (no; yes) and smoking during pregnancy (no; yes). We included in multivariable models, child’s age and sex, maternal age, parity, Raven score, education, marital status, monthly household income, and alcohol and smoking during pregnancy.

### Statistical analysis

Chlordecone and other chemicals’ concentrations were log-transformed (base 2) to address skewness and limit the influence of outliers. Values below limit of detection were replaced by LOD/$\surd 2$ [17]. Initial exploratory analyses included descriptive statistics and univariate associations between exposures and measured outcomes, and potential covariates of interest. We also investigated correlations between log-transformed concentrations of environmental exposures using Pearson correlations.

**Fig. 1:**
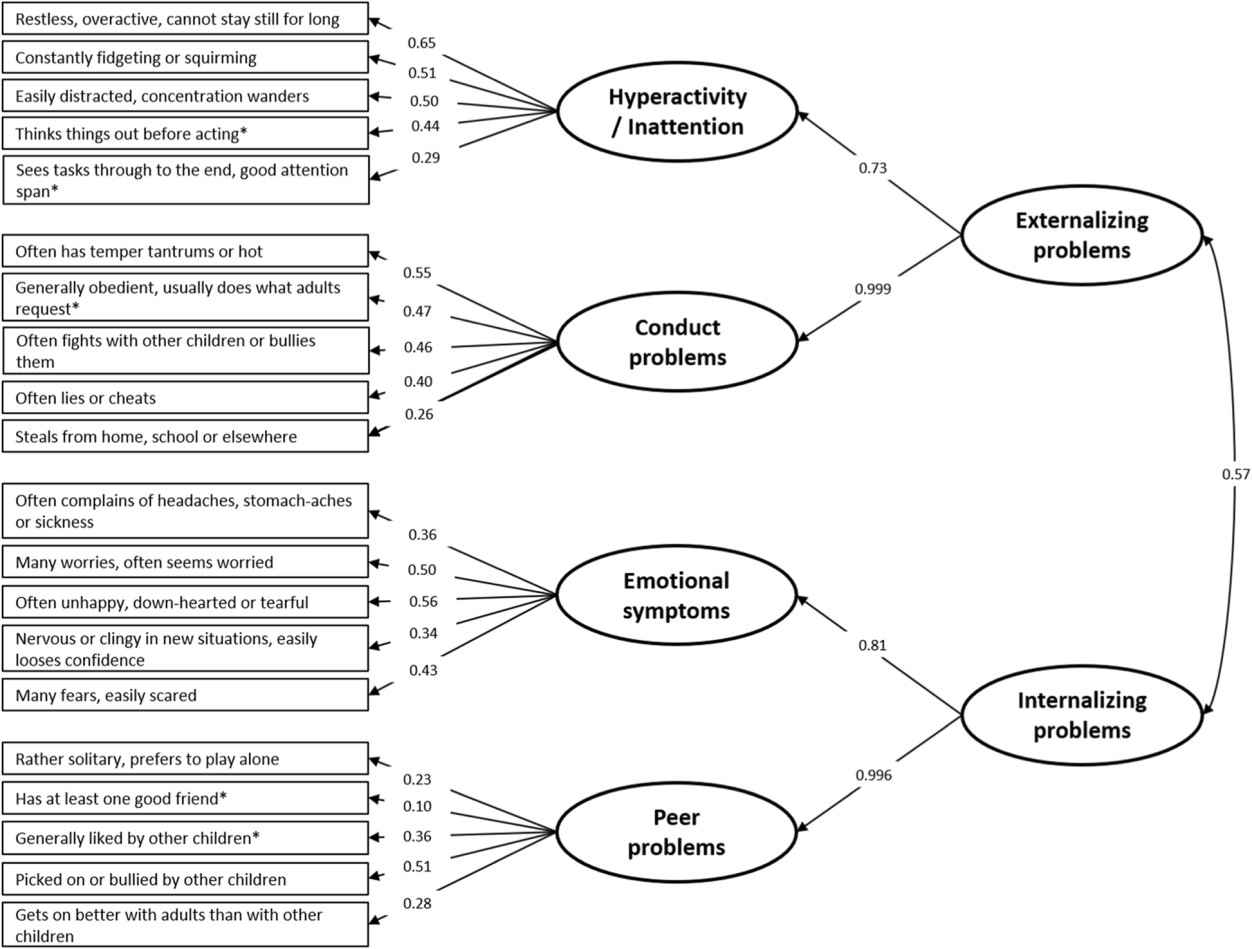
Conceptual diagram of the confirmatory factor analysis for the behavioral domain. Numbers in the arrows indicate the percent variance in the scores explained by the higher order latent functions, whereas numbers in double-headed arrows indicate covariances (All factor loadings were significant at *p* < 0.001). Items with an asterisk (*) were scored reversely

First, we used structural equations modeling (SEM) adjusting for the same set of covariates to investigate simultaneously the associations between cord- and 7-years exposures, with WISC-IV and SDQ scores. We ran two separate analyses for the cognitive and behavioral functions. For the WISC-IV scores, we employed a simple path analysis to incorporate all outcomes in a single model. The results are presented as the absolute change in the test score associated with a twofold increase in chlordecone concentrations. For the SDQ test, we used a two stages confirmatory factor analysis (CFA) model with first order latent functions of conduct problems, hyperactivity/inattention, emotional symptoms, and peer problems indicated by their subsequent measured SDQ item scores (Fig. 1). We added two second order latent functions for externalizing problems (indicated by conduct problems and hyperactivity/inattention first order latent functions) and internalizing problems (indicated by emotional symptoms, and peer problems first order latent functions) (Fig. 1). We adopted this two-stage CFA model since it has previously been found to show superior model fits and because it was considered theoretically meaningful [26]. All observed indicators showed significant correlations to their latent functions (Fig. 1), and the models exhibited an excellent fit to the data (Comparative fit index = 0.92, root mean square error of approximation = 0.04, Standardized Root Mean Square Residual = 0.035, and χ^2^ *p*-value: 0.06). For the SDQ SEM model, the results for behavioral functions are presented as a standard deviation (SD) change in the latent functions associated with a twofold increase in chlordecone concentrations. The SEM approach allows to reduce both multiple comparisons testing and measurement errors. Because there were missing data for some covariates and prenatal chlordecone exposures, we used the Full Information Maximum Likelihood estimation, which utilizes all available information and avoids list-wise deletion due to missing data.

We also examined potential effect modification by sex using multi-group SEM analyses. In these analyses, we constrained the loadings, variances, co-variances, and intercepts of the latent variables to be equal across the two groups of females and males. Differences in the associations in the two exposure groups were tested by comparing the value of *d*/SE_*d*_ to the standard normal distribution, where *d* is the difference between the two estimates, and ${SE}_{d}=\sqrt{{SE}_{1}^{2}+{SE}_{2}^{2}}$ is the standard error of the difference [1, 28].

In additional analyses, we conducted multivariable linear regression analyses to investigate the associations between cord- and 7-years chlordecone concentrations in relation to the WISC-IV (FSIQ and four composite scores). The results are presented as the adjusted mean difference (aMD) in scores with 95% confidence interval (95% CI) for a two-fold increase in chlordecone concentrations. For the SDQ internalizing and externalizing measured scores, and because scores on these tests exhibit an over-dispersed distribution, we used negative binomial regressions. Negative binomial regressions model the ratio of the mean SDQ scores among exposed and non-exposed. The results are presented as adjusted mean ratios (aMR) of the SDQ externalizing and internalizing problems with corresponding 95% CIs. In all these models, the exposure estimate was reported for a two-fold increase in chlordecone concentrations—for example, an aMR of 1.1 for a two-fold increase in chlordecone suggests that the mean SDQ subscale score was 10% higher for each doubling of the chlordecone concentrations. We also examined sex-specific associations by including a product interaction term (sex x chlordecone concentrations) in all analyses.

We finally explored potential nonlinear relations of chlordecone concentrations (log2-transformed) with WISC-IV and SDQ scores using generalized additive models (GAMs) with penalized smoothing regression splines [40] and visually inspected plots of the smoothed data. For the behavioral function (externalizing problems and internalizing problems), we extracted the latent function scores from the factor-analytic component of the SEM and used them as outcome variables, adjusting for covariates [27]. Cord-blood and 7-years child chlordecone concentrations were introduced as spline functions. We assessed departure from linearity (at *p* < 0.1) by comparing the model with chlordecone concentrations introduced as a spline function with chlordecone concentrations introduced as a linear term. None of the dose–response relationships significantly deviated from linearity, all at a *p* > 0.2 (Supplemental Material; Figures S3 and S4).

In sensitivity analyses, we adjusted simultaneously for additional exposures measured in cord blood that have been shown to exert neurotoxic effects, namely, blood Pb, Cd, and Hg, and lipid-standardized PCB-153 and *pp’*-DDE concentrations.

All significance tests were two-sided, and the level of significance was set at a *p*-value < 0.05 for main estimates and interactions. However, we provide all the estimates, and base our conclusions on the effect estimates in addition to concordant trends rather than solely on hypothesis testing. All statistical analyses were conducted using R version 4.0.3 [38].

## Results

The characteristics of the study population are presented in Table 1. Outcome scores and 7-years chlordecone concentrations were available for 446 children whereas only 371 children had information on both outcome scores and cord chlordecone concentrations. About half were boys (49%) and mean age at examination was 7.6 years. Mean maternal age at delivery was 32 years, most of the mothers were born in the French West Indies and more than a half achieved a high school diploma or college/university studies. About 34% of mothers were nulliparous, and 40% of children were breastfed for more than 6 months. Overall, children included in the prenatal and 7-years analyses did not differ with the sample of children followed up at the 7-year examination (Table 1).

**Table 1:** Description of important characteristics of the study population

**Characteristic**	All (*n* = 576)		With data available in both child blood and outcomes at 7-years (*n* = 446)		With data available in both cord blood and 7-years outcomes (*n* = 371)	
	n	Mean (SD) or n (%)	n	Mean (SD) or n (%)	n	Mean (SD) or n (%)
**Child characteristics**						
Sex	576		446		371	
Male		279 (48.4%)		217 (48.6%)		170 (45.8%)
Female		297 (51.6%)		229 (51.4%)		201 (54.2%)
Child age (years)	576	7.7 (0.2)	446	7.6 (0.2)	371	7.7 (0.2)
Birth weight (g)	576	3117.9 (542.7)	446	3128.7 (530.9)	371	3141.5 (515.4)
**Maternal Characteristics**						
Maternal age at delivery (years)	576	31.9 (6.6)	446	32.1 (6.5)	371	31.7 (6.7)
Parity	576		446		371	
Nulliparous		200 (34.7%)		150 (33.6%)		131 (35.3%)
Primipara		191 (33.2%)		151 (33.9%)		121 (32.6%)
Multiparous		185 (32.1%)		145 (32.5%)		119 (32.1%)
Breastfeeding duration (months)	576		446		371	
No breastfeeding		90 (15.6%)		67 (15.0%)		60 (16.2%)
≤ 6 months		258 (44.8%)		201 (45.1%)		158 (42.6%)
7 – 18 months		119 (20.7%)		99 (22.2%)		81 (21.8%)
≥ 18 months		109 (18.9%)		79 (17.7%)		72 (19.4%)
Maternal marital status	576		446		371	
Married or in a couple		314 (56.0%)		238 (55.1%)		207 (57.7%)
Single		140 (25.0%)		114 (26.4%)		85 (10.2%)
Living with own family		106 (19.0%)		80 (18.5%)		69 (32.1%)
Missing		16		14		10
Maternal education	576		446		371	
None or elementary school		26 (4.5%)		21 (4.7%)		17 (4.6%)
Some high school		275 (47.8%)		213 (47.8%)		172 (46.4%)
High school diploma		124 (21.5%)		98 (22.0%)		91 (24.5%)
College/University studies		151 (26.2%)		114 (25.5%)		91 (24.5%)
Household income (euros)	566		437		364	
≤ 800 Euros		55 (9.7%)		44 (10.1%)		33 (9.2%)
800 – 2300 Euros		295 (52.1%)		233 (53.3%)		181 (50.7%)
> 2300 Euros		216 (38.2%)		160 (36.6%)		143 (40.1%)
Missing		10		9		7
Maternal Raven score	541	35.4 (12.2)	420	35.3 (12.1)	345	35.4 (12.4)
Maternal smoking during pregnancy	576		446		371	
No		558 (96.9%)		434 (97.3%)		359 (96.8%)
Yes		18 (3.1%)		12 (2.7%)		12 (3.2%)
Alcohol during pregnancy	547		425		361	
Never		535 (97.8%)		416 (97.9%)		351 (97.2%)
Ever		12 (2.2%)		9 (2.1%)		10 (2.8%)
Missing		29		21		10
Neurodevelopmental outcomes						
WISC-IV composite scores						
FSIQ	569	87.1 (16.8)	442	86.6 (16.6)	369	87.1 (17.0)
Verbal comprehension	569	93.8 (16.2)	442	93.3 (16.1)	369	93.7 (16.0)
Perceptive reasoning	569	83.9 (19.1)	442	83.4 (19.0)	369	84.3 (19.5)
Processing speed	569	91.8 (15.2)	442	91.7 (15.1)	369	91.7 (15.0)
Working memory	569	92.1 (15.1)	442	91.8 (15.0)	369	91.5 (15.2)
SDQ subscales						
Internalizing problems	576	4.7 (3.3)	452	4.4 (3.2)	371	4.9 (3.3)
Externalizing problems	576	7.0 (4.0)	452	7.0 (4.1)	371	6.9 (4.0)

*SD* Standard deviation, *SDQ* Strengths and difficulties questionnaire, *FSIQ* Full-scale IQ, *WISC-IV* Wechsler intelligence scale for children, fourth edition

Table 2 shows the distribution of environmental chemicals in cord and child samples. Chlordecone was detected in 88% and 83% of cord and child blood samples, respectively. The median chlordecone concentration in cord blood (0.21 μg/L) was higher than the one observed in child blood samples (0.05 μg/L). The percentage of detected values for prenatal and postnatal exposure to other chemicals were all greater than 80% except for cord PCB-153 and cord Cd where 70% and 53% of the values were detected, respectively. Figure S3 shows the correlation heat map between multiple chemicals in cord and 7-years blood. The intercorrelation between cord and child chlordecone concentrations was very low (*r* = 0.02), as are the correlation coefficients between cord chlordecone and other contaminants assessed in cord blood (*r*’s range from -0.14 to 0.13), and between child chlordecone and other child chemicals (*r*’s range from -0.17 to 0.30). The correlations between and within other chemicals at different time points were weak to moderate, with the highest correlations observed between cord and 7-years pp’-DDE (*r* = 0.34).

**Table 2:** Distribution of environmental contaminants in the study population (µg/L)

**Time point**	**Exposure**	**n**	**% < LOD**							
				**Min**	**25**^**th**^	**50**^**th**^	**Mean**	**75**^**th**^	**95**^**th**^	**Max**
Cord	Chlordecone	369	12%	< LOD	0.07	0.21	0.57	0.38	1.55	29.8
	PCB-153	368	30%	< LOD	< LOD	0.06	0.12	0.15	0.48	1.75
	pp'-DDE	368	7.4%	< LOD	0.10	0.28	0.67	0.69	2.71	12.5
	Lead	391	0%	5.2	10.2	13.3	15.6	18.2	30.7	81.0
	Mercury	391	0%	0.8	4.4	6.6	7.3	9.3	15.0	46.1
	Cadmium	391	47%	< LOD	< LOD	0.05	0.10	0.11	0.35	0.91
7-years	Chlordecone	442	17%	< LOD	0.02	0.05	0.12	0.11	0.37	7.01
	PCB-153	442	8.3%	< LOD	0.03	0.07	0.10	0.12	0.32	1.29
	pp'-DDE	442	1.9%	< LOD	0.09	0.19	0.47	0.41	1.40	26.4
	Lead	438	0%	7.9	16.3	22.4	23.2	27.4	37.9	213
	Mercury	438	0%	0.2	1.0	1.7	2.2	2.80	5.2	19
	Cadmium	438	32%	< LOD	< LOD	0.10	0.12	0.18	0.25	0.79

Table S1 shows the univariate associations of our primary measured outcomes (FSIQ, SDQ internalizing and externalizing problems scores) with important characteristics of the study population. FSIQ scores were associated with child sex, birth weight, maternal age, parity, marital status, education, household income, breastfeeding duration, and maternal Raven scores. SDQ internalizing problems scores were higher in children from mothers that are younger, single, that have a lower education, household income, and Raven score. Lower birth weight was also associated with higher internalizing problems scores. SDQ externalizing problems scores were associated with the same characteristics in addition to child sex with higher scores in boys.

### Associations between cord and 7-years chlordecone concentrations and scores of cognitive abilities

**Fig. 2:**
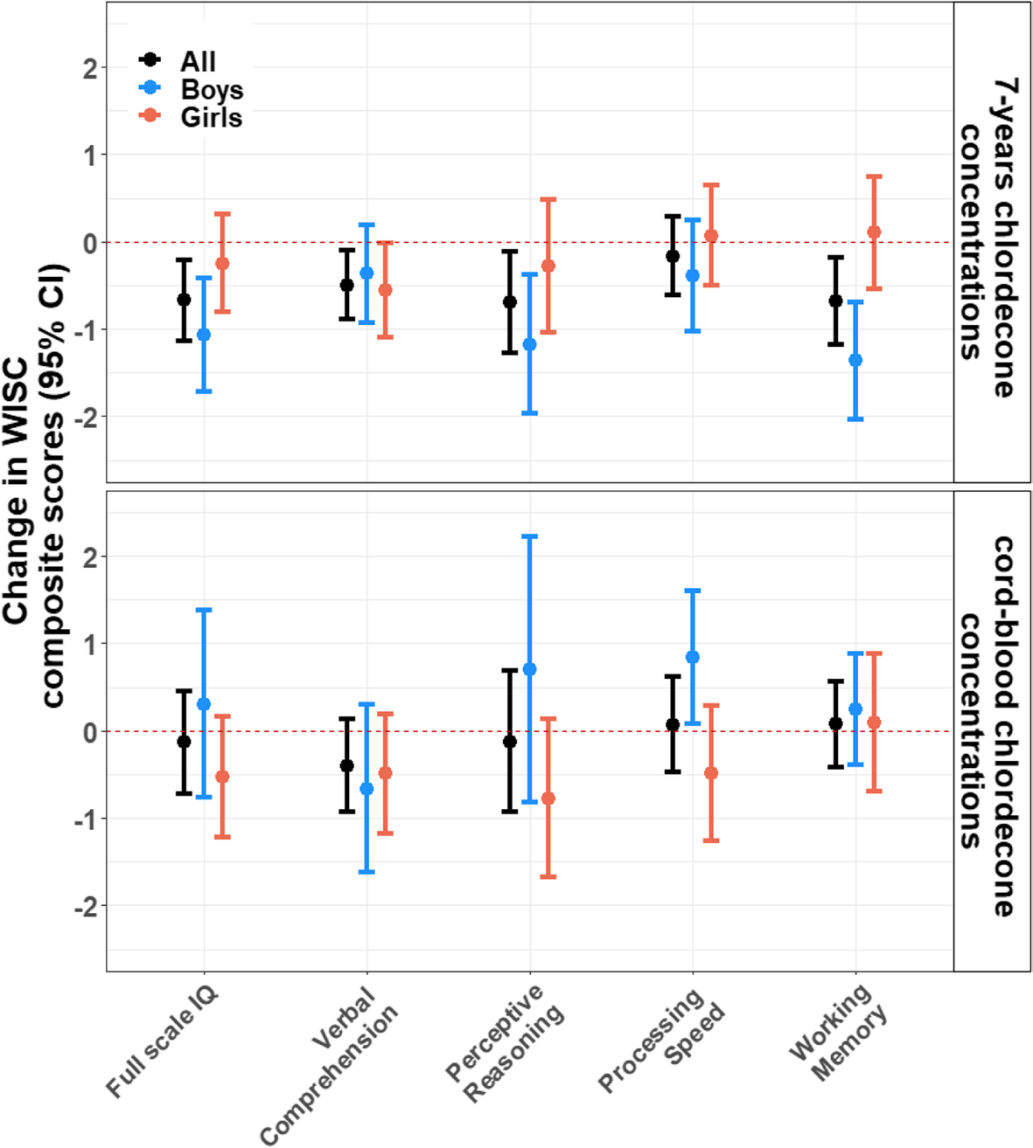
Associations between cord-blood and 7-years chlordecone concentrations and WISC-IV composite scores obtained from the SEM path analysis, stratified by sex. Models were adjusted for child’s age and sex, maternal age, parity, Raven score, education, marital status, monthly household income, and alcohol and smoking during pregnancy

Results of these analyses are presented in Fig. 2 and Supplementary material, Table S2. A twofold increase in 7-year chlordecone concentrations was associated with 0.67 (95% CI: -1.13, -0.22) lower FSIQ scores. This association was driven by 7-years chlordecone associations with working memory (β = -0.69; 95% CI: -1.18, -0.19), verbal comprehension (β = -0.50; 95% CI: -0.89, -0.10), and perceptive reasoning composite scores (β = -0.69; 95% CI: -1.27, -0.11). No association was observed with processing speed (β = -0.17; 95% CI: -0.61, 0.28). In multiple-group SEM sex-stratified analyses, a few associations showed effect modification by sex at p-effect modification (p-EM) < 0.05. Associations between 7-year chlordecone concentrations and some WISC-IV composite scores were stronger in boys (Fig. 2). For instance, a twofold increase in 7-years chlordecone concentrations was associated with 1.07 (95% CI: -1.72, -0.42) and 1.36 (95% CI: -2.04, -0.69) lower FSIQ and working memory scores in boys whereas the association was weaker or null in girls (β = -0.25; 95% CI: -0.81, 0.31 and β = 0.10; 95% CI: -0.54, 0.75 for FSIQ and working memory, respectively; p-EM = 0.06 and 0.002, respectively). Additionally, cord blood chlordecone concentrations were associated with 0.84 (95% CI: 0.08, 1.60) higher processing speed scores in boys whereas the association was negative in girls (β = -0.48; 95% CI: -1.26, 0.29; p-EM = 0.02). No effect modification by sex was observed for other scores of cognitive abilities.

### Associations between cord and 7-years chlordecone concentrations and behavioral problems

**Fig. 3:**
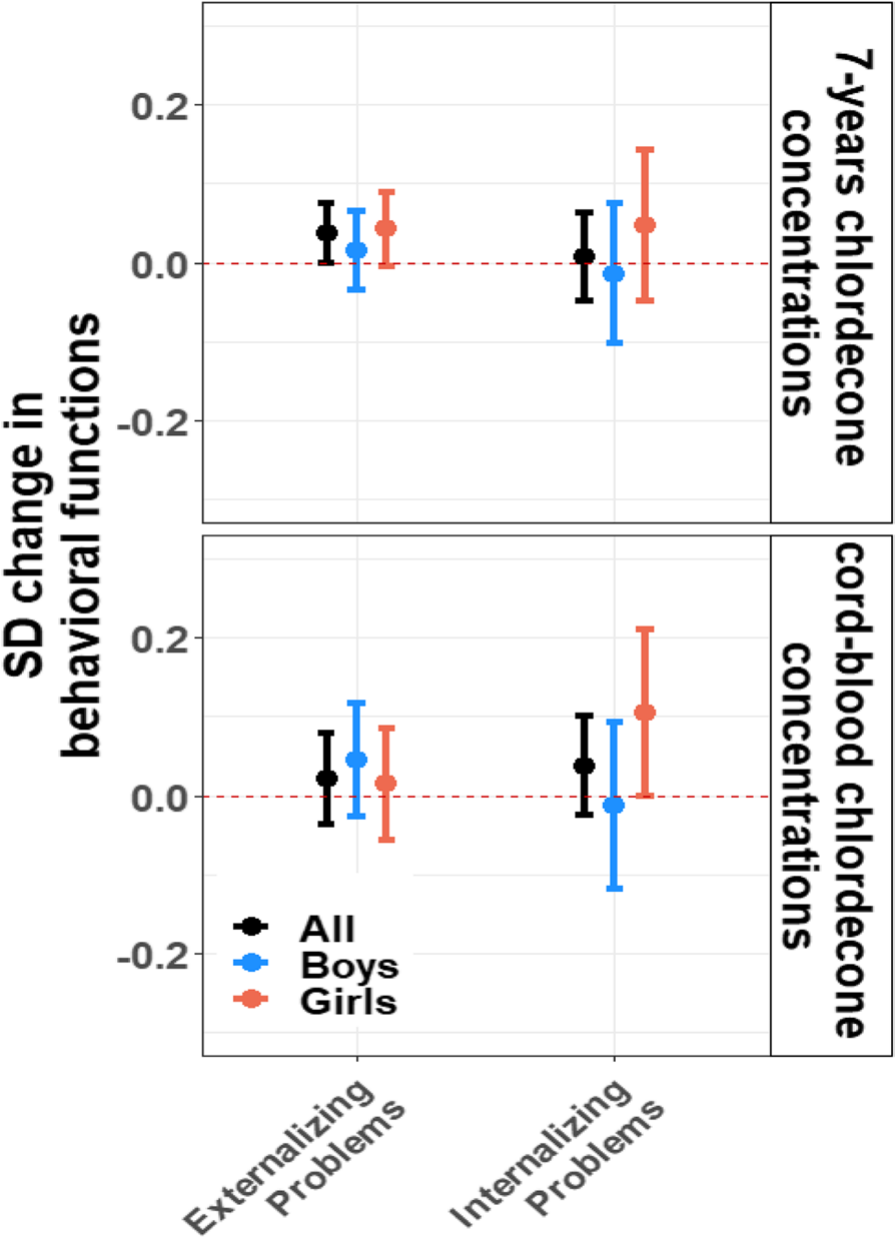
Associations between cord-blood and 7-years chlordecone concentrations and standardized behavioral functions obtained using SEM analyses, stratified by sex. Models were adjusted for child’s age and sex, maternal age, parity, Raven score, education, marital status, monthly household income, and alcohol and smoking during pregnancy

Results of these analyses are presented in Fig. 3 and Supplementary material, Table S2. Prenatal exposure to chlordecone was not associated with externalizing (B = 0.02 SD; 95% CI: -0.04, 0.08) or internalizing (B = 0.04 SD; 95% CI: -0.02, 0.10) problems. A twofold increase in 7-years chlordecone concentrations was associated with 0.04 SD (95% CI: 0.0, 0.08) higher (worse) externalizing problems scores. No association was observed between 7-years chlordecone concentrations and internalizing problems (B = 0.01 SD; 95% CI: -0.05, 0.06). We also did not observe any effect modification by sex in multiple-group SEM analyses (all at p-EM > 0.10).

### Additional analyses

**Fig. 4:**
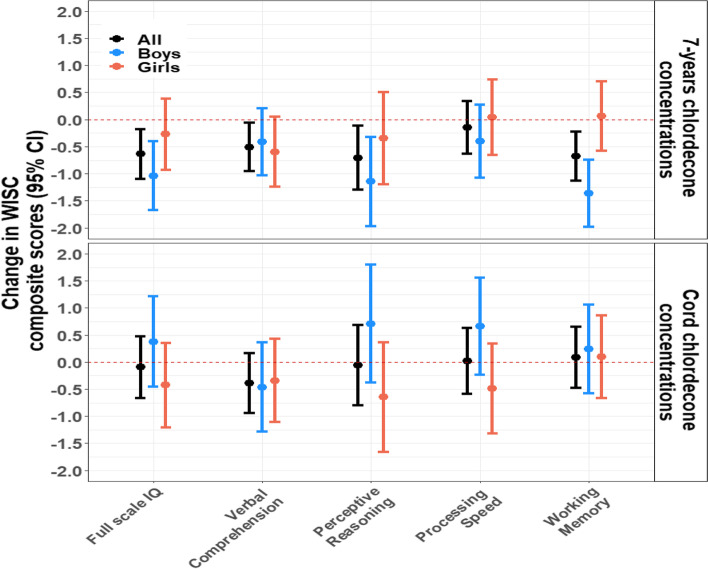
Associations between cord-blood and 7-years chlordecone concentrations and WISC-IV composite scores using multivariable linear analyses, stratified by sex. Models were adjusted for child’s age and sex, maternal age, parity, Raven score, education, marital status, monthly household income, and alcohol and smoking during pregnancy

**Fig. 5:**
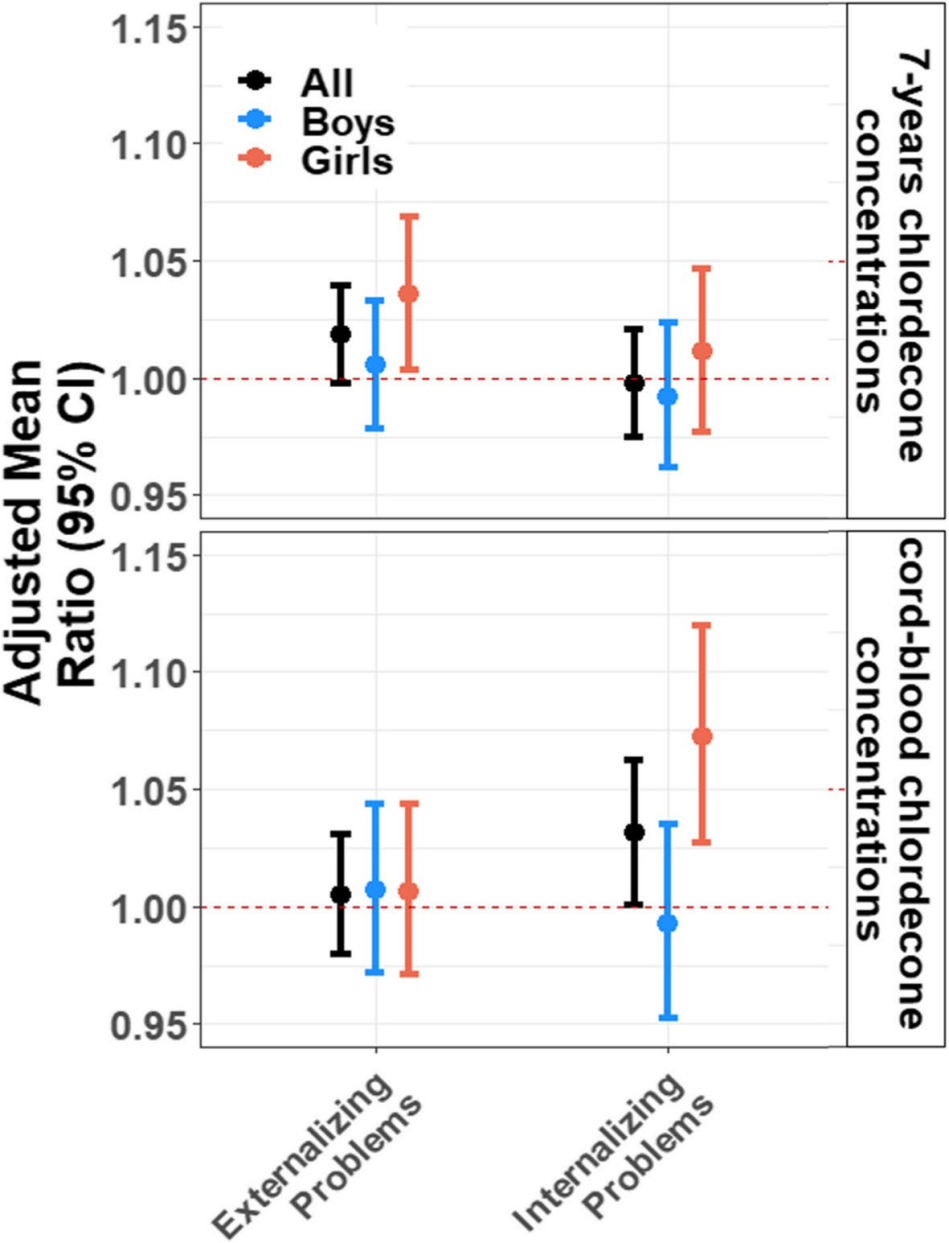
Associations between cord-blood and 7-years chlordecone concentrations and measured SDQ scores using multivariable negative binomial models, stratified by sex. Models were adjusted for child’s age and sex, maternal age, parity, Raven score, education, marital status, monthly household income, and alcohol and smoking during pregnancy

In additional analyses using traditional multivariable regressions, we observed similar patterns: analyses showed concordant findings for both cognitive scores using linear models (Fig. 4) and behavioral scores using negative binomial models (Fig. 5). For instance, a twofold increase in 7-years chlordecone concentrations was associated with 0.64 (95% CI: -1.09, -0.18) lower FSIQ scores. Similar results were observed for perceptive reasoning, verbal comprehension, and working memory scores. We also observed stronger associations in boys than in girls for working memory as reported in the SEM analyses.

For the SDQ scores, a twofold increase in cord-blood chlordecone concentrations was associated with 3% higher internalizing problems scores (aMR = 1.03; 95% CI: 1.00, 1.06), with an effect modification by sex where associations were stronger in girls (aMR = 1.07; 95% CI: 1.03, 1.12) than in boys (aMR = 0.99; 95% CI: 0.95, 1.04). A twofold increase in 7-years chlordecone concentrations was also associated with 2% higher externalizing problems scores (aMR = 1.02; 95% CI: 1.00, 1.04; Fig. 5).

Analyses investigating non-linear significant dose–response relationships showed no significant departure from linearity for any of the investigated associations (Supplemental Material; Figures S4 and S5). Additional sensitivity analyses adjusting for other co-exposures showed similar findings (Supplemental material; Figures S6 and S7).

## Discussion

We previously reported effects in domains of cognitive and fine motor functions of pre- and postnatal exposures to chlordecone in 7- and 18-months old children from the TIMOUN cohort. However, there is currently no empirical data to determine the degree to which prenatal and postnatal chlordecone exposures are associated with effects in school-aged children. In the present study, we investigated both pre- and postnatal exposures to chlordecone in relation to cognitive abilities and problem behaviors at age 7 years. Our findings point to a potential detrimental effect of 7-years chlordecone concentrations, but not from in utero exposure, on cognitive abilities, with stronger effects in boys. These effects are seen with general cognitive abilities as measured by the WISC-IV, but also in specific nonverbal domains of perceptive reasoning and working memory, and verbal comprehension. Regarding behavioral problems, higher chlordecone exposure at 7 years of age was associated with higher scores of externalizing problems, while prenatal chlordecone exposure was related to higher internalizing problems scores, with stronger associations among girls. Patterns of results are very robust, with significant associations consistently repeated when outcome scores are obtained from structural equations modeling or analyzed traditionally using multivariable regressions.

When aged 7-months old, infants from this cohort exhibited poorer visual recognition memory or novelty preference in relation with pre- and postnatal chlordecone exposure. Prenatal exposure was also associated with slower visual processing speed and poorer fine motor development at 7-months [9] and at 18-months in boys only [5]. In a recent investigation from the same cohort, we also reported that 7-years chlordecone concentrations were associated with poorer visual processing when copying geometric figures using the Stanford Binet copying test, while no association was observed with cord chlordecone concentrations [11].

In animal studies, chlordecone was shown to alter catecholamine activity—including dopamine—by decreasing their synaptic binding and uptake [10]. Additionally, male rats exposed to chlordecone were found to be hypersensitive to the motility increasing effects of apomorphine, a dopamine receptor agonist [37]. Chlordecone has also been shown to possess estrogen-like activity [16, 23] which may mediate the observed neurotoxic effects, especially those pertaining to the hypothalamo-pituitary axis [10]. This may also explain some sex-specific associations observed in this study. For instance, chlordecone exposure during the critical period for sexual differentiation of the brain has been shown to alter sex-dependent behaviors in adult rats [22].

Our results point to potential sexually dimorphic effects of chlordecone on both cognitive and behavioral functions. Prenatal chlordecone exposure was related to higher internalizing problems scores, with stronger associations in girls, whereas postnatal exposures exhibited stronger effects in boys, especially for working memory. In a previous investigation from this cohort, higher cord chlordecone concentrations were associated with an increase in thyroid stimulating hormone in 3-month boys, whereas postnatal exposure concentrations in breastmilk were associated with a decrease in free triiodothyronine overall, and in free thyroxine among girls [7]. Similar sexually dimorphic associations were observed in this cohort with fine motor skills at 18 months [5] and visual contrast sensitivity at 7-years [33].

Several other organochlorine insecticides have been found to have sex-specific associations, both in animal studies and in human studies [21]. For instance, perinatal exposure of mice during gestation and lactation to dieldrin altered dopaminergic neurochemistry and had greater adverse effects in the male offspring than the female [30]. Other organochlorine insecticides showed opposite effects with stronger effects on girls. For instance, prenatal exposure to *pp’*-DDE showed stronger associations with behavioral problems at age 7–8 years in girls [36], whereas maternal Dichlorodiphenyltrichloroethane (DDT) and *pp’*-DDE serum concentrations were associated with processing speed at 7-years of age, with *pp’*-DDE associations exhibiting stronger estimates in girls [12]. The current levels of chlordecone measured in this cohort show a significant decrease from a geometric mean of 0.13 µg/L in cord blood to 0.06 µg/L at 7-year-old children with no differences by child sex.

Despite the low levels observed in this cohort, especially at 7 years, we were still able to detect associations with specific domains of development. Interestingly, prenatal Hg was also associated with poorer performance in same domains of cognitive function, including both perceptual reasoning and verbal comprehension in Faroese children [14] as in Inuit children from Northern Canada where both cohorts were exposed through maternal consumption of contaminated fish and mammals [19]. Similar domains have also been shown to be vulnerable to other neurotoxicants (Reviewed in [35] and these specific domains of cognitive function might be more sensitive to neurotoxic chemicals. Similar to other investigations, the observed effect sizes in this study are relatively modest and subtle. However, given the widespread and ubiquitous exposure to chlordecone in this population, these small effect sizes could had a considerable impact at the population level [3].

This study has several notable strengths. We use a prospective longitudinal cohort, with enrollment of mothers during their pregnancy, collection of biological samples for assessment of multiple exposures at different time points, multiple maternal interviews to document covariates, and long-term follow-up of a large number of children assessed with gold standard tests to assess cognitive function independently from maternal report. Additionally, we assessed both prenatal and postnatal exposures through state-of-the-art laboratory analyses, concurrently with assessment of other chemicals recognized or under study for their neurotoxicity such as Pb, Cd, Hg, PCB-153 and *pp’*-DDE. We were also able to document and measure important covariates that allowed us to thoroughly assess potential confounders, including several socioeconomic status indicators, as well as maternal non-verbal cognitive abilities. The significant associations are reported between child Full-Scale IQ and variables such as breastfeeding, family income, maternal marital status, education and Raven scores confirm the critical role of SES and family environment in promoting child development. Furthermore, obtaining these significant associations with variables consistently related to child cognitive skills and behavior in studies conducted in multiple populations, provides convergent validation for use of the WISC-IV and the SDQ in Guadeloupean children. Finally, we used a SEM approach, therefore addressing issues arising from multiple testing and missing data that may not be adequately considered by standard regression analyses. Comparison of the SEM findings with traditional approaches analyzing each test score separately yielded comparable findings.

There are several limitations to this study. The reported associations could be attributable to unmeasured confounders, and although we cannot rule out possible residual or additional unmeasured confounding by other factors, we adopted a conservative approach by adjusting for several indicators of SES, i.e. marital status, income, and maternal education, even if they lay on a common causal pathway, thus reducing the potential for residual confounding. Our results are also unlikely to be attributable to other contaminants since we have adjusted for several neurotoxicants in sensitivity analysis even though their correlations with chlordecone concentrations were weak or null. The significant cross-sectional associations at 7-years limits the ability to draw strong causal inferences, although reverse causality is improbable as we do not expect cognitive and behavioral functions in the child to influence their dietary behaviors, the main source of exposure in the general population in Guadeloupe. Indeed, dietary choices at this age are mainly directed by the parent.

Despite the ban of chlordecone since the 90 s, it persists in soils for decades if not centuries, constituting a long-lasting source of exposure to future generations. It has also been shown recently that chlordecone fluxes in soils drastically increased when glyphosate use began, leading to widespread ecosystem contamination [32]. The use of glyphosate in Guadeloupe may therefore lead to an increase in soil erosion and release of the stable chlordecone stored in the soils of polluted fields.

## Conclusion

This is the first study to examine effects of chlordecone on cognitive and behavioral functions in school age children. The results from this study suggest that concurrent exposures to chlordecone may be associated with child development in specific domains of cognition, especially in boys, whereas prenatal exposures may be associated with behavioral function in girls. This study provides new insights on the potential neurotoxicity of chlordecone, which persists in the FWI population decades after its ban.

## Supplementary Information

**Additional file 1:** **Figure S1.** Flow chart of the Timoun study (Neurodevelopment at seven years). **Figure S2.** Conceptual Directed Acyclic Graph representing the associations between chlordecone exposure, FSIQ, and potential confounders. **Figure S3.** Correlation plot of maternal and 7-years exposures. **Table S1.** Univariate associations between primary outcomes and characteristics of the study population. **Table S2.** Associations between cord- and 7-years blood chlordecone concentrations and cognitive and behavioral functions using SEM analyses, stratified by sex. **Figure S4.** Dose response relationship between cord- and 7-years blood chlordecone concentrations and WISC-IV scores. **Figure S5.** Dose response relationship between cord- and 7-years blood chlordecone concentrations and behavioral latent functions. **Figure S6.** Associations between cord-blood and 7-years chlordecone concentrations and WISC-IV composite scores obtained from the SEM path analysis with additional adjustment for co-exposures, stratified by sex. **Figure S7.** Associations between cord-blood and 7-years chlordecone concentrations and standardized behavioral functions obtained using SEM analyses with additional adjustment for co-exposures, stratified by sex.

## Data Availability

The analyses for this study were performed at the University of Massachusetts Amherst and University of Laval. The datasets used in this study are not publicly available due to participant confidentiality but are available from the corresponding author on reasonable request following appropriate human subjects training and IRB approval.
