# Supplementary material for: Prenatal and childhood chlordecone exposure, cognitive abilities and problem behaviors in 7-year-old children: the TIMOUN mother–child cohort in Guadeloupe
Source: Environ Health. 2023 Feb 27;22:21. doi: 10.1186/s12940-023-00970-3 (PMC9969702; doi:10.1186/s12940-023-00970-3)
Supplement: Supplementary file 1 — Additional file 1: Figure S1. Flow chart of the Timoun study (Neurodevelopment at seven years). Figure S2. Conceptual Directed Acyclic Graph representing the associations between chlordecone exposure, FSIQ, and potential confounders. Figure S3. Correlation plot of maternal and 7-years exposures. Table S1. Univariate associations between primary outcomes and characteristics of the study population. Table S2. Associations between cord- and 7-years blood chlordecone concentrations and cognitive and behavioral functions using SEM analyses, stratified by sex. Figure S4. Dose response relationship between cord- and 7-years blood chlordecone concentrations and WISC-IV scores. Figure S5. Dose response relationship between cord- and 7-years blood chlordecone concentrations and behavioral latent functions. Figure S6. Associations between cord-blood and 7-years chlordecone concentrations and WISC-IV composite scores obtained from the SEM path analysis with additional adjustment for co-exposures, stratified by sex. Figure S7. Associations between cord-blood and 7-years chlordecone concentrations and standardized behavioral functions obtained using SEM analyses with additional adjustment for co-exposures, stratified by sex. [file 12940_2023_970_MOESM1_ESM.docx]

**Supplemental Material. Early life exposure to Chlordecone and cognitive and behavioral outcomes in Guadeloupian Children**

**Table of content:**

**Figure S1.** Flow chart of the Timoun study (Neurodevelopment at seven years)

**Figure S2.** Conceptual Directed Acyclic Graph representing the associations between chlordecone exposure, FSIQ, and potential confounders

**Figure S3.** Correlation plot of maternal and 7-years exposures

**Table S1.** Univariate associations between primary outcomes and characteristics of the study population.

**Table S2.** Associations between cord- and 7-years blood chlordecone concentrations and cognitive and behavioral functions using SEM analyses, stratified by sex.

**Figure S4.** Dose response relationship between cord- and 7-years blood chlordecone concentrations and WISC-IV scores.

**Figure S5.** Dose response relationship between cord- and 7-years blood chlordecone concentrations and behavioral latent functions.

**Figure S6.** Associations between cord-blood and 7-years chlordecone concentrations and WISC-IV composite scores obtained from the SEM path analysis with additional adjustment for co-exposures, stratified by sex.

**Figure S7.** Associations between cord-blood and 7-years chlordecone concentrations and standardized behavioral functions obtained using SEM analyses with additional adjustment for co-exposures, stratified by sex.


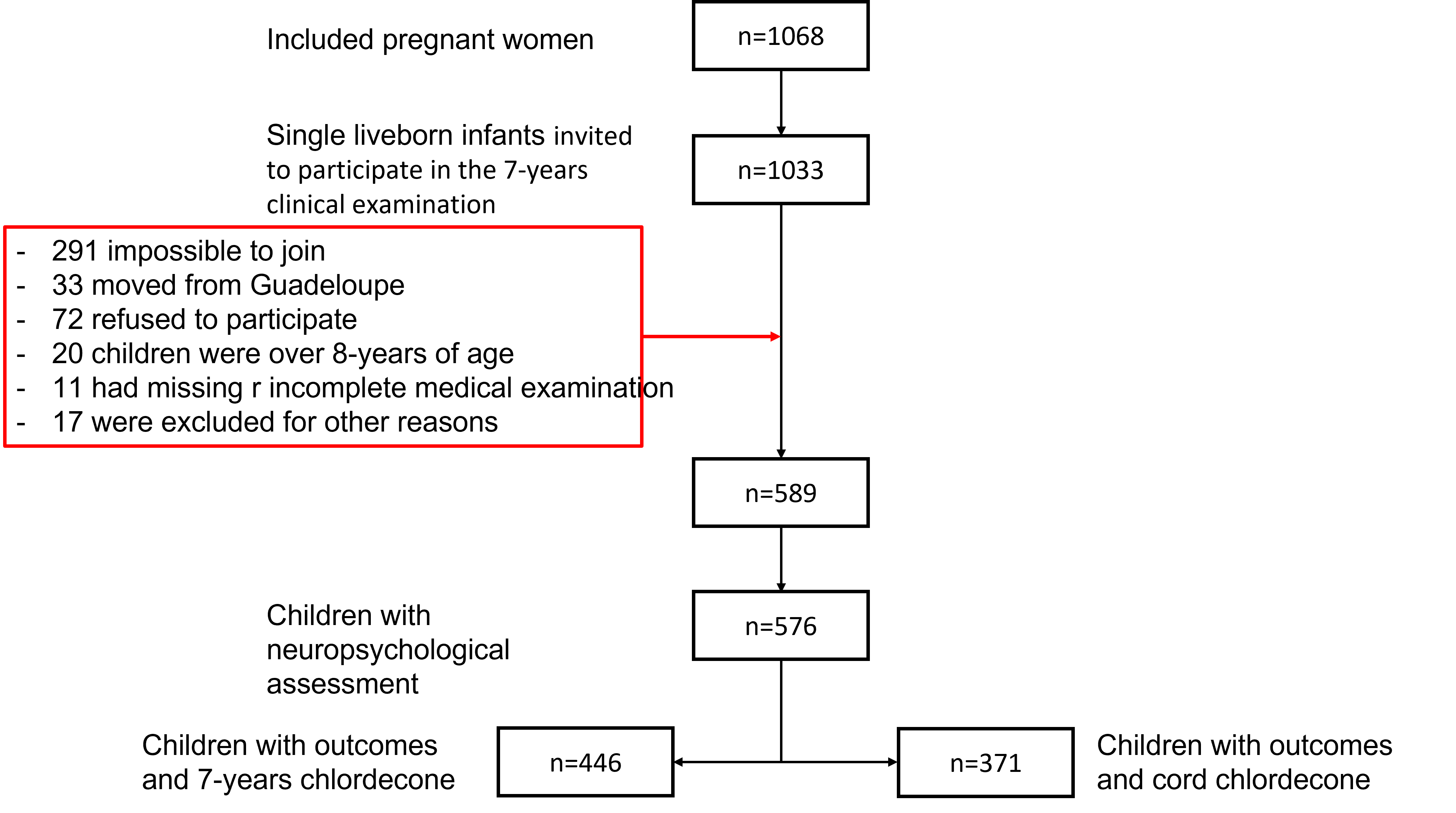


**Figure S1.** Flow chart of the Timoun study (Neurodevelopment at seven years)


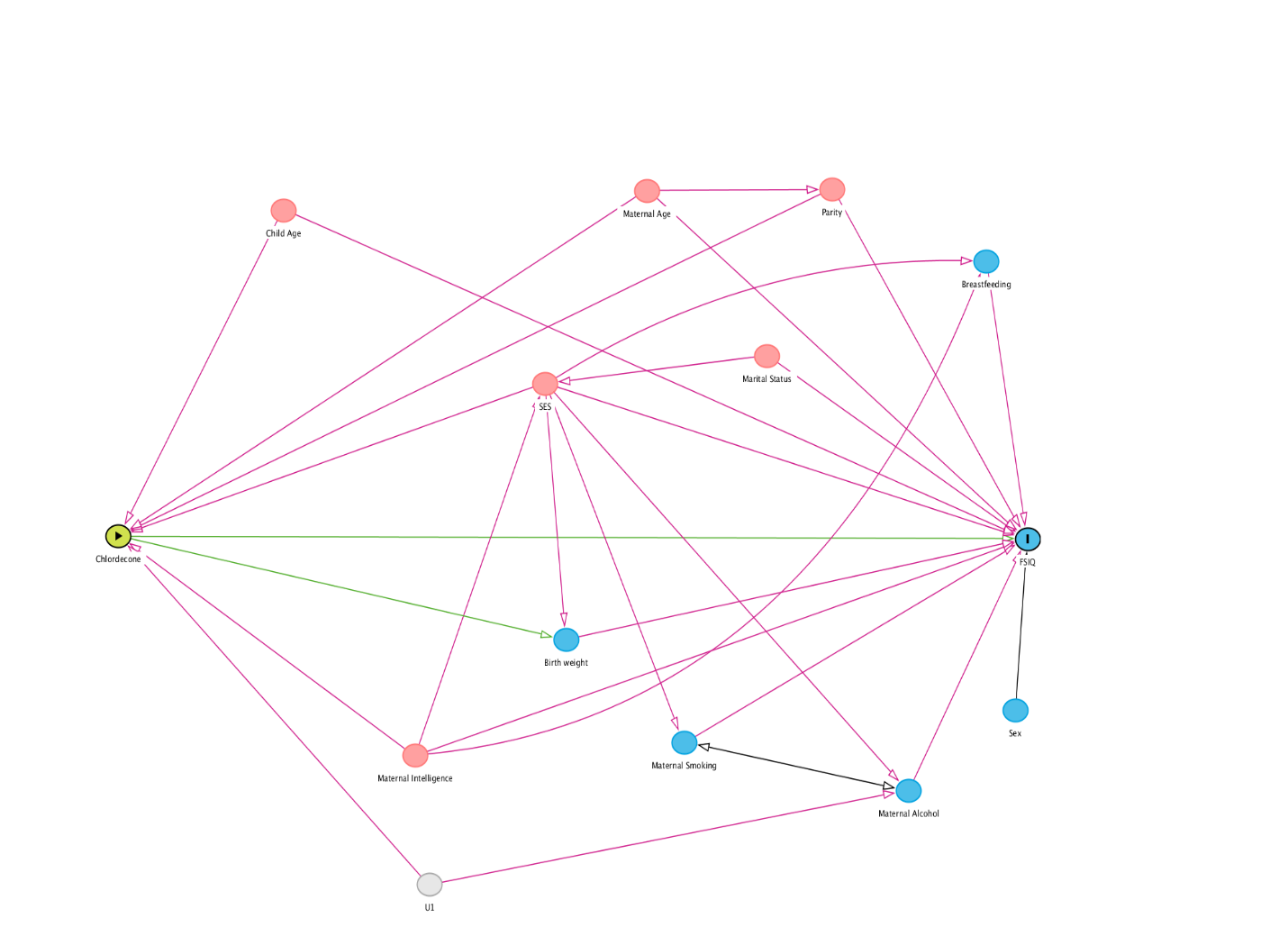


**Figure S2.** Conceptual Directed Acyclic Graph representing the associations between chlordecone exposure, FSIQ, and potential confounders


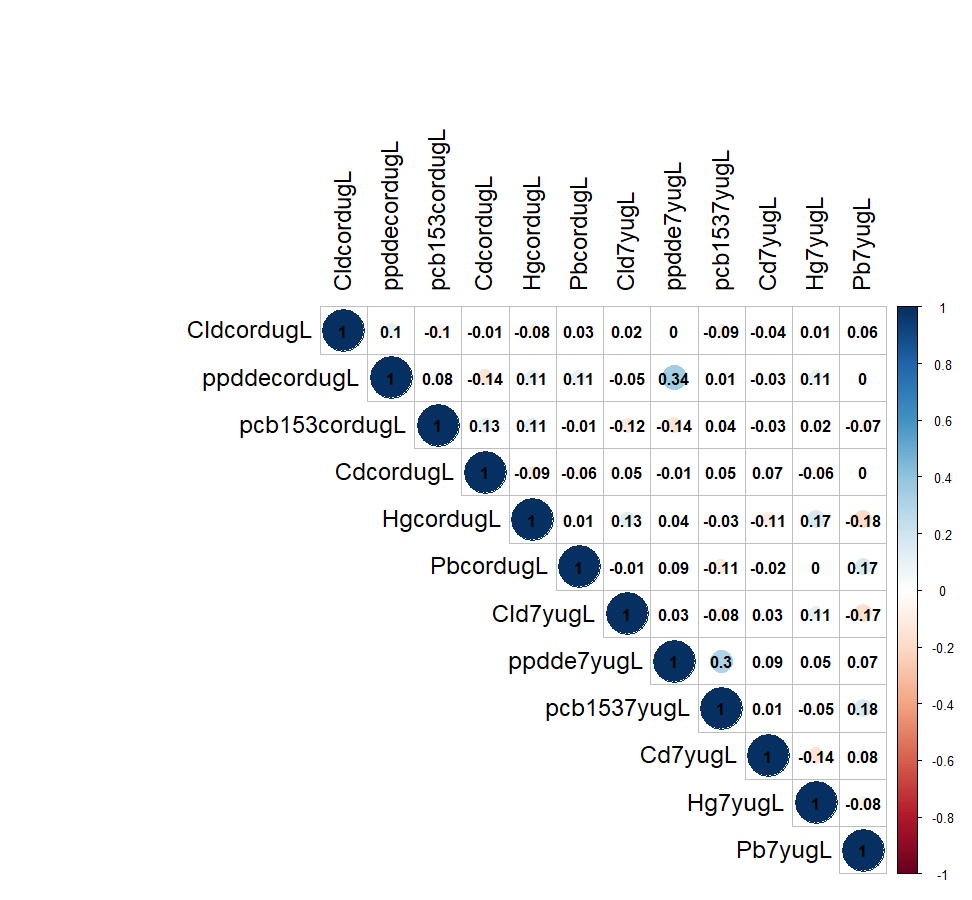


**Figure S3.** Correlation plot of cord and 7-years exposures. Cd: Cadmium; Cld: Chlordecone; Hg: Mercury; Pb: Lead; pp-DDE: pp- dichlorodiphenyl dichloroethane; PCB: dichlorodiphenyl dichloroethane.

Table S1. Univariate associations between primary outcomes and characteristics of the study population.

| **Characteristic** | n | FSIQ | | Internalizing problems scores | | Externalizing problems scores | |
| --- | --- | --- | --- | --- | --- | --- | --- |
|  |  | Mean  (SE) | p-value | Mean (SE) | p-value | Mean  (SE) | p-value |
| **Child characteristics** |  |  |  |  |  |  |  |
| Sex | 446 |  | 0.02 |  | 0.39 |  | <0.001 |
| Male | 217 | 85.0 (1.2) |  | 4.5 (3.3) |  | 8.4 (4.0) |  |
| Female | 229 | 88.1 (1.0) |  | 4.3 (3.0) |  | 5.6 (3.6) |  |
| Child age (years) | 446 |  | 0.75 |  | 0.67 |  | 0.88 |
| < 7.8 years | 283 | 86.5 (1.0) |  | 4.3 (3.1) |  | 7.0 (4.1) |  |
| 7.8 years | 163 | 86.7 (1.3) |  | 4.5 (3.3) |  | 6.9 (4.1) |  |
| Low Birth weight (< 2500g) | 446 |  | 0.03 |  | 0.02 |  | 0.03 |
| No | 394 | 87.2 (0.8) |  | 4.3 (3.1) |  | 6.8 (4.0) |  |
| Yes | 52 | 81.9 (2.3) |  | 5.3 (3.6) |  | 8.1 (4.4) |  |
| **Maternal Characteristics** |  |  |  |  |  |  |  |
| Maternal age at delivery | 446 |  | <0.001 |  | 0.07 |  | 0.02 |
| < 25 years | 73 | 79.5 (1.9) |  | 5.2 (3.0) |  | 7.3 (4.0) |  |
| 25 - 35 years | 210 | 87.3 (1.1) |  | 4.3 (3.2) |  | 7.4 (4.1) |  |
| 35 years | 163 | 88.8 (1.2) |  | 4.2 (3.2) |  | 6.2 (3.9) |  |
| Parity | 446 |  | 0.002 |  | 0.09 |  | 0.57 |
| Nulliparous | 150 | 88.0 (1.5) |  | 4.6 (3.1) |  | 7.2 (4.2) |  |
| Primipara | 151 | 87.8 (1.3) |  | 4.7 (3.5) |  | 6.7 (3.9) |  |
| Multiparous | 145 | 83.9 (1.2) |  | 3.9 (2.9) |  | 7.0 (4.1) |  |
| Breastfeeding duration |  |  | <0.001 |  | 0.35 |  | 0.45 |
| No breastfeeding | 67 | 81.4 (2.2) |  | 4.5 (3.2) |  | 7.4 (4.1) |  |
| ≤ 6 months | 201 | 87.3 (1.1) |  | 4.6 (3.2) |  | 7.1 (4.2) |  |
| 7 - 18 months | 99 | 91.2 (1.7) |  | 4.3 (3.4) |  | 6.6 (3.8) |  |
| ≥ 18 months | 79 | 83.2 (1.7) |  | 3.9 (2.8) |  | 6.6 (3.9) |  |
| Maternal marital status | 446 |  | <0.001 |  | <0.001 |  | <0.001 |
| Married or in a couple | 238 | 89.5 (1.0) |  | 4.0 (3.0) |  | 6.5 (3.9) |  |
| Single | 114 | 85.0 (1.5) |  | 4.7 (3.5) |  | 7.3 (4.0) |  |
| Living with own family | 80 | 79.3 (1.8) |  | 4.8 (3.7) |  | 7.5 (4.2) |  |
| Missing | 14 | - |  | - |  | - |  |
| Maternal education | 446 |  | <0.001 |  | <0.001 |  | 0.006 |
| None or elementary school | 21 | 75.7 (3.1) |  | 5.6 (3.5) |  | 9.1 (4.3) |  |
| Some high school | 213 | 81.7 (1.0) |  | 4.7 (3.1) |  | 7.3 (4.0) |  |
| High school diploma | 98 | 89.2 (1.8) |  | 4.6 (3.1) |  | 6.9 (4.2) |  |
| College/University studies | 114 | 95.5 (1.3) |  | 3.3 (3.0) |  | 6.1 (3.9) |  |
| Household income (euros) | 437 |  | <0.001 |  | <0.001 |  | 0.03 |
| ≤ 800 Euros | 44 | 78.6 (2.1) |  | 5.3 (2.5) |  | 7.3 (4.2) |  |
| 800 – 2300 Euros | 233 | 82.9 (1.0) |  | 4.7 (3.3) |  | 7.3 (4.1) |  |
| >2300 Euros | 160 | 94.1 (1.3) |  | 3.6 (3.0) |  | 6.3 (3.8) |  |
| Missing | 9 | - |  | - |  | - |  |
| Maternal Raven score | 420 |  | <0.001 |  | 0.07 |  | 0.004 |
| < 37 | 207 | 79.7 (1.0) |  | 4.7 (3.2) |  | 7.6 (4.2) |  |
| ≥ 37 | 213 | 92.6 (1.1) |  | 4.1 (3.1) |  | 6.5 (4.0) |  |
| Missing | 26 | - |  | - |  | - |  |
| Maternal smoking during pregnancy | 446 |  | 0.73 |  | 0.22 |  | 0.60 |
| No | 434 | 86.4 (0.8) |  | 4.4 (3.2) |  | 7.0 (4.1) |  |
| Yes | 12 | 92.8 (3.6) |  | 5.5 (2.3) |  | 7.6 (3.8) |  |
| Alcohol during pregnancy | 425 |  | 0.20 |  | 0.89 |  | 0.76 |
| Never | 416 | 86.5 (0.8) |  | 4.4 (2.4) |  | 7.0 (4.1) |  |
| Ever | 9 | 92.6 (4.8) |  | 4.1 (3.7) |  | 6.6 (4.7) |  |
| Missing | 21 | - |  | - |  | - |  |

Table S2. Associations between cord- and 7-years blood chlordecone concentrations and cognitive and behavioral functions using SEM analyses, stratified by sex. Models were adjusted for child’s age and sex, maternal age, parity, Raven score, education, marital status, monthly household income, and alcohol and smoking during pregnancy.

| **Test** | **Function** | **All** | | **Sex-stratified Estimates (95% CI)** | | **P-effect modification** |
| --- | --- | --- | --- | --- | --- | --- |
|  |  | **Estimate**  **(95% CI)** | **p-value** | **Boys** | **Girls** |  |
| **Cord Blood** | | | | | | |
| SDQ | Internalizing problems | 0.04 (-0.02, 0.10) | 0.23 | 0.00 (-0.07, 0.07) | 0.10 (0.01, 0.19) | 0.08 |
|  | Externalizing problems | 0.02 (-0.04, 0.08) | 0.46 | 0.04 (-0.06, 0.13) | 0.01 (-0.06, 0.09) | 0.72 |
| WISC-IV | Working memory | 0.07 (-0.42, 0.56) | 0.78 | 0.24 (-0.39, 0.88) | 0.09 (-0.70, 0.88) | 0.76 |
|  | Perceptive reasoning | -0.12 (-0.93, 0.69) | 0.77 | 0.70 (-0.82, 2.22) | -0.75 (-1.66, 0.15) | 0.11 |
|  | Processing speed | 0.07 (-0.47, 0.61) | 0.79 | 0.84 (0.08, 1.60) | -0.48 (-1.26, 0.29) | 0.02 |
|  | Verbal comprehension | -0.40 (-0.93, 0.14) | 0.15 | -0.66 (-1.63, 0.18) | -0.48 (-1.17, 0.20) | 0.77 |
|  | Full scale IQ | -0.13 (-0.71, 0.45) | 0.66 | 0.31 (-0.76, 1.38) | -0.53 (-1.22, 0.16) | 0.20 |
| **7 Years Blood** | | | | | | |
| SDQ | Internalizing problems | 0.01 (-0.05, 0.06) | 0.78 | -0.01 (-0.07, 0.04) | 0.02 (-0.03, 0.08) | 0.36 |
|  | Externalizing problems | 0.04 (0.00, 0.08) | 0.04 | 0.03 (-0.03, 0.08) | 0.05 (0.01, 0.10) | 0.43 |
| WISC-IV | Working memory | -0.69 (-1.18, -0.19) | 0.01 | -1.36 (-2.04, -0.69) | 0.10 (-0.54, 0.75) | 0.002 |
|  | Perceptive reasoning | -0.69 (-1.27, -0.11) | 0.02 | -1.18 (-1.97, -0.38) | -0.28 (-1.04, 0.47) | 0.11 |
|  | Processing speed | -0.17 (-0.61, 0.28) | 0.46 | -0.39 (-1.02, 0.24) | 0.07 (-0.50, 0.64) | 0.29 |
|  | Verbal comprehension | -0.50 (-0.89, -0.10) | 0.01 | -0.37 (-0.93, 0.19) | -0.56 (-1.10, -0.02) | 0.62 |
|  | Full scale IQ | -0.67 (-1.13, -0.22) | 0.003 | -1.07 (-1.72, -0.42) | -0.25 (-0.81, 0.31) | 0.06 |

**
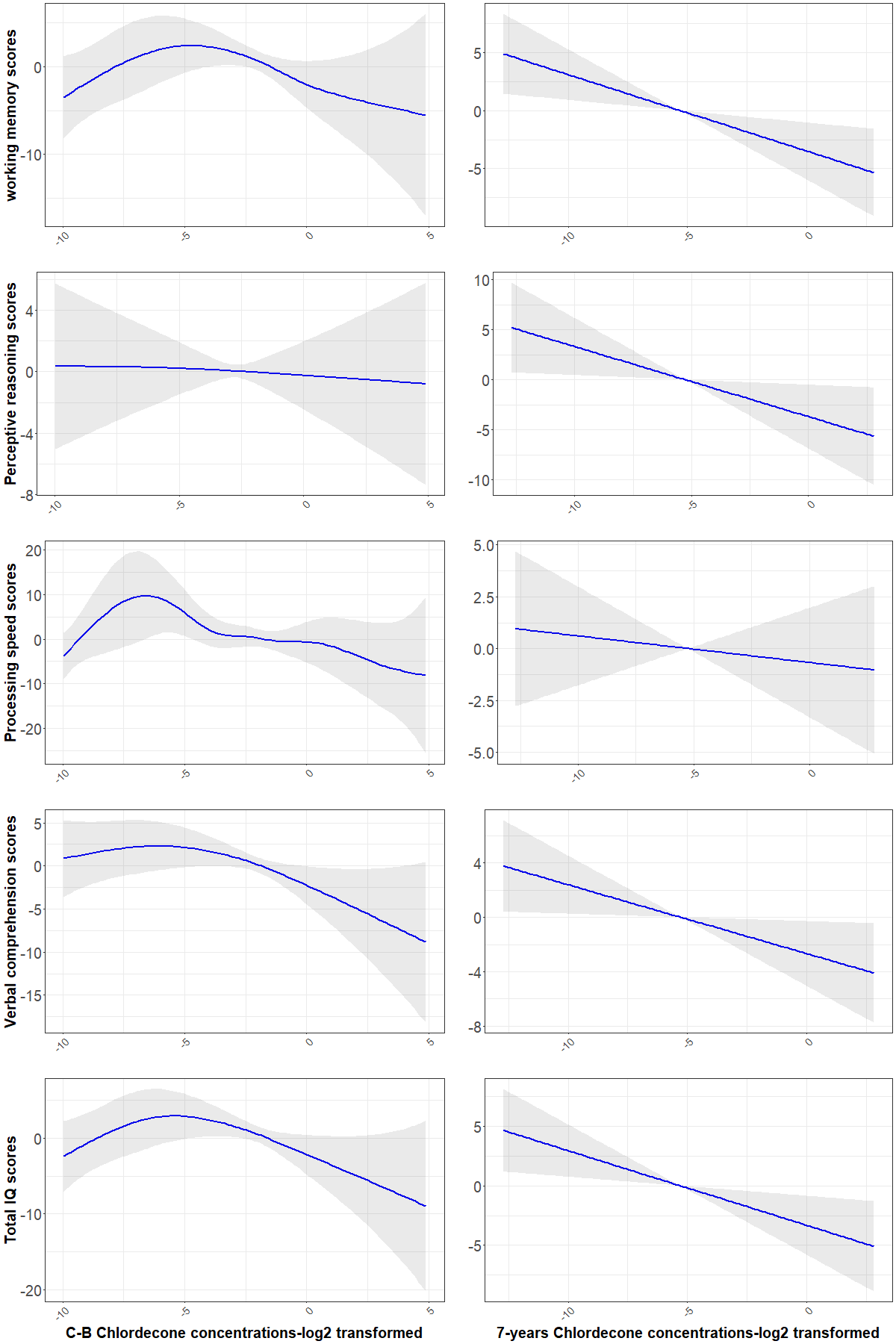
**

**Figure S4.** Dose response relationship between cord- and 7-years blood chlordecone concentrations and WISC-IV scores.


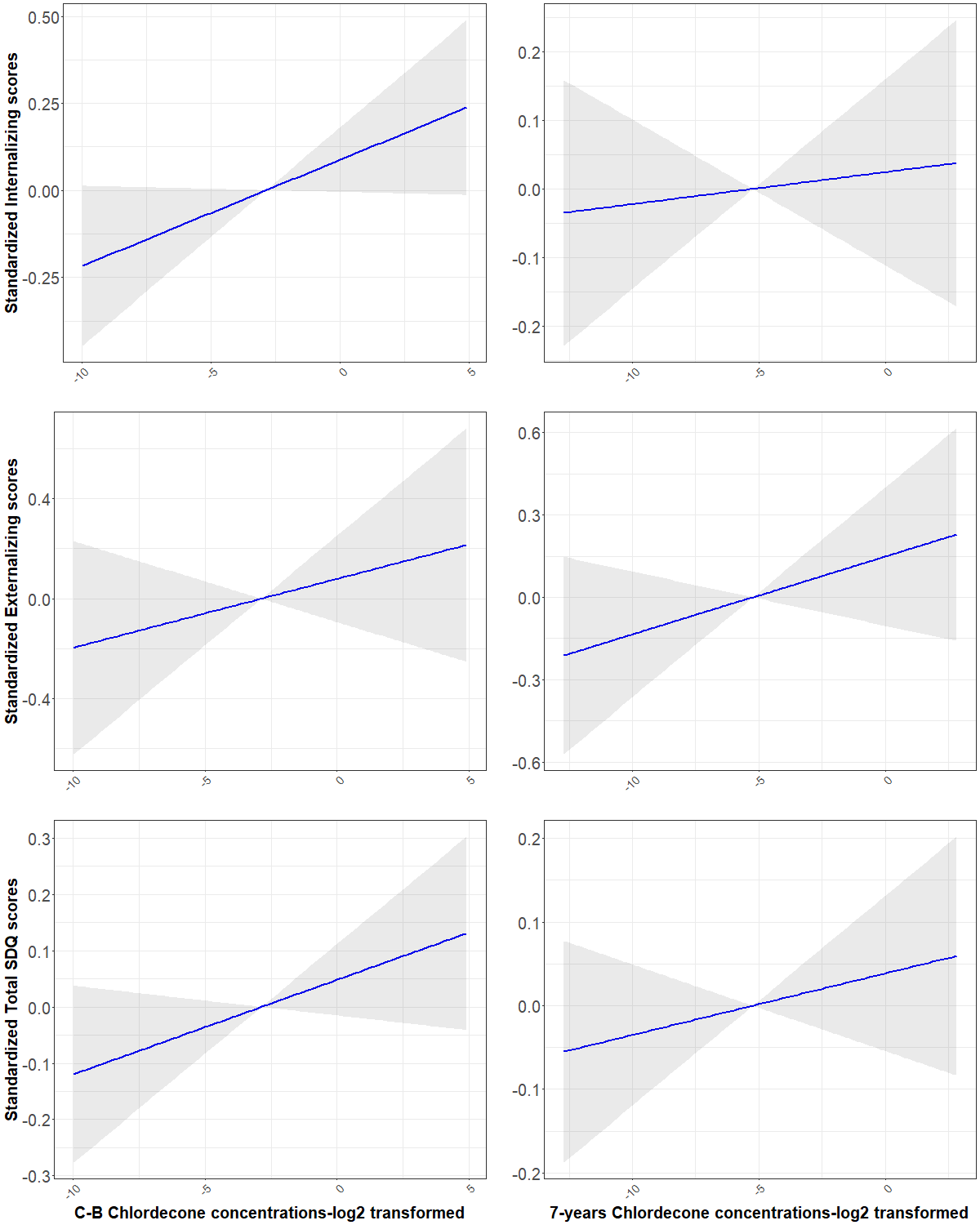

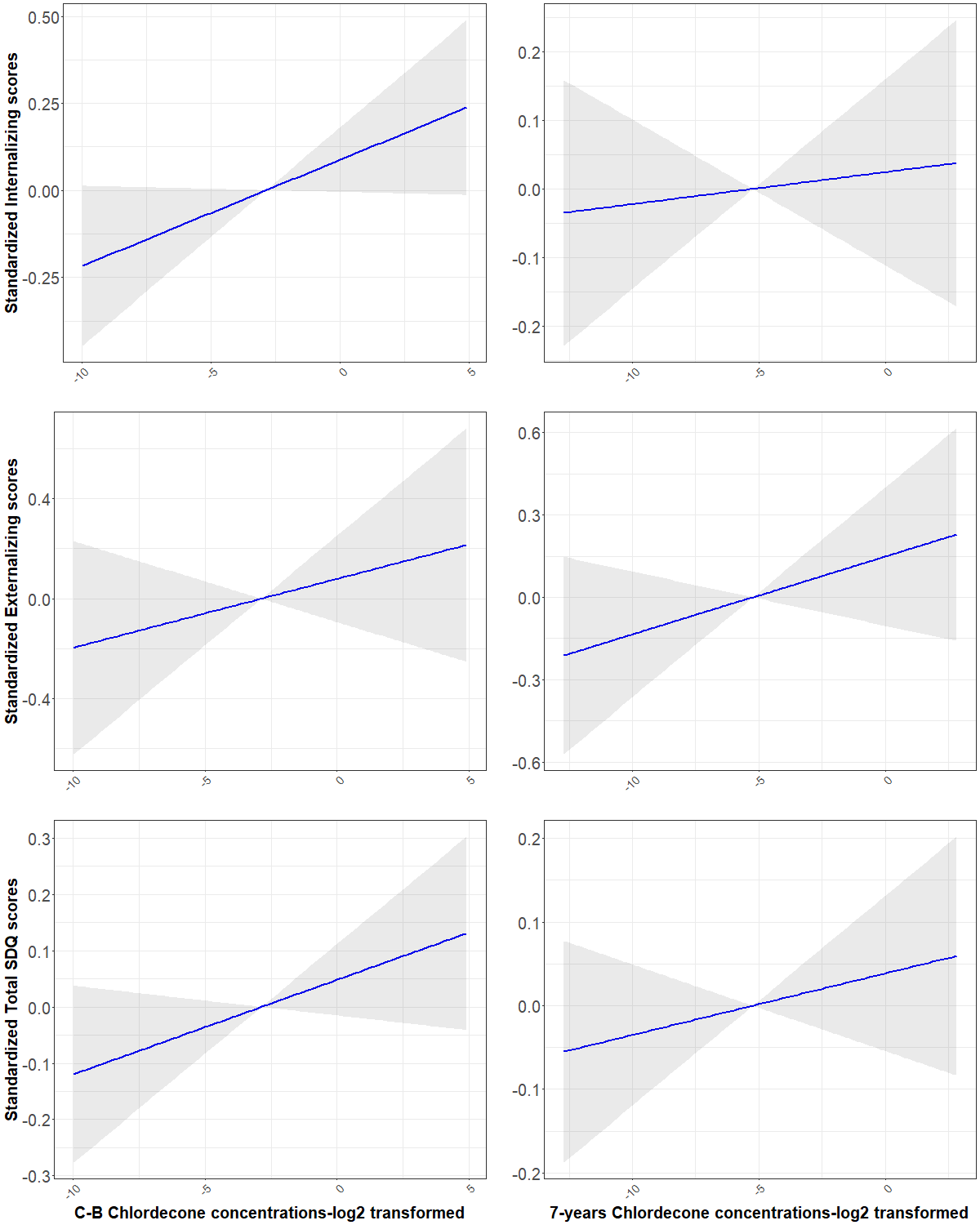


**Figure S5.** Dose response relationship between cord- and 7-years blood chlordecone concentrations and behavioral latent functions.


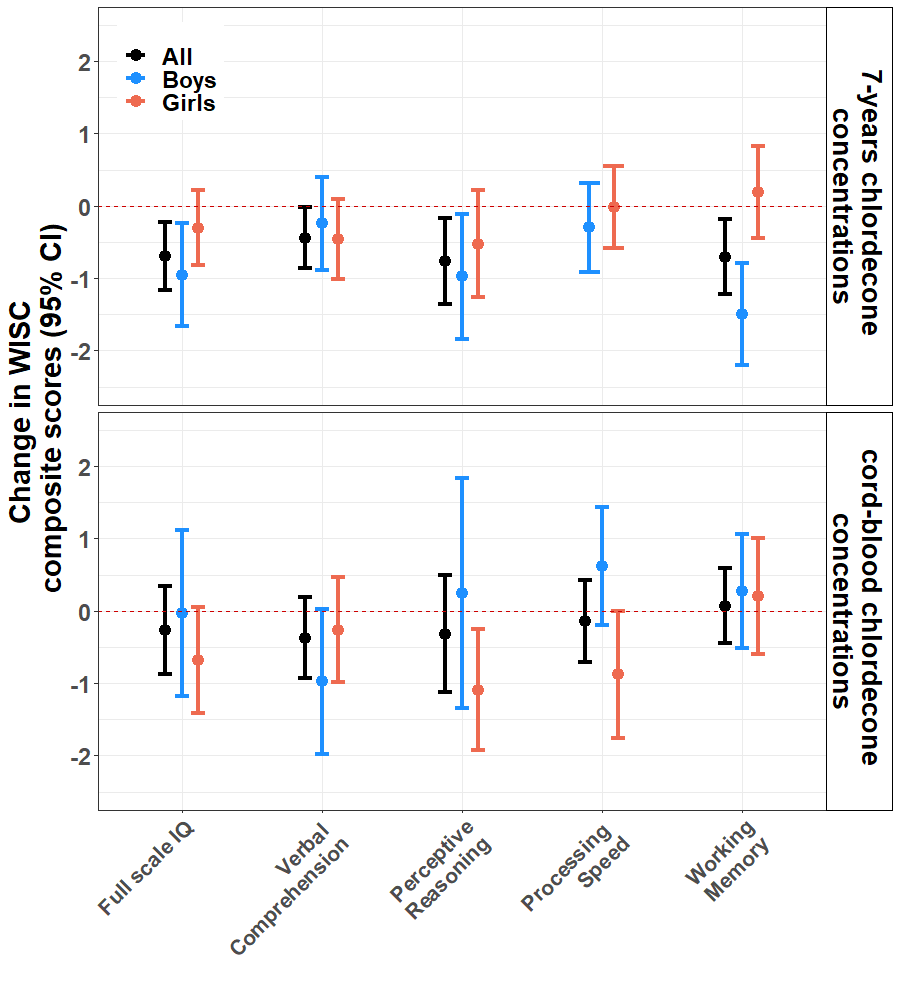


**Figure S6**. Associations between cord-blood and 7-years chlordecone concentrations and WISC-IV composite scores obtained from the SEM path analysis with additional adjustment for co-exposures (i.e. Pb, Cd, Hg, PCB-153, and pp-DDE), stratified by sex. Models were adjusted for child’s age and sex, maternal age, parity, Raven score, education, marital status, monthly household income, and alcohol and smoking during pregnancy, in addition to the co-environmental exposures.


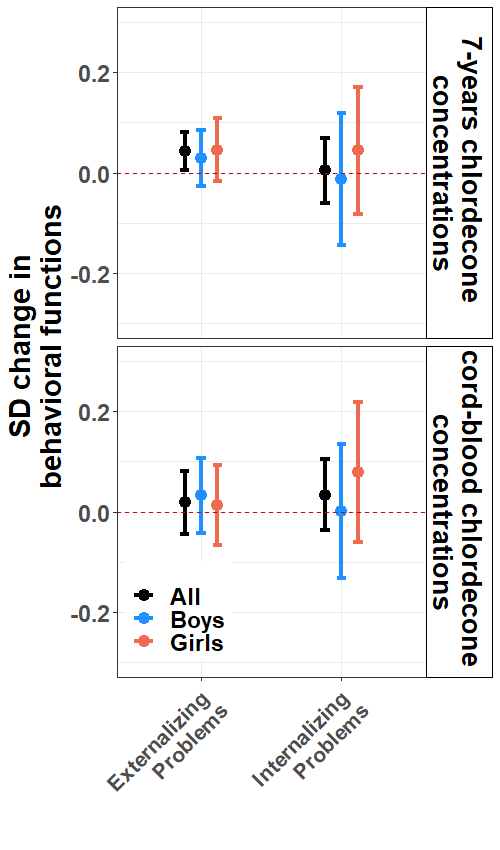


**Figure S7.** Associations between cord-blood and 7-years chlordecone concentrations and standardized behavioral functions obtained using SEM analyses with additional adjustment for co-exposures (i.e. Pb, Cd, Hg, PCB-153, and pp-DDE), stratified by sex. Models were adjusted for child’s age and sex, maternal age, parity, Raven score, education, marital status, monthly household income, and alcohol and smoking during pregnancy, in addition to the co-environmental exposures.
